# Effects of weaning and castration ages on growth performance, blood metabolites, and carcass characteristics in Hanwoo steers

**DOI:** 10.1186/s40781-018-0188-2

**Published:** 2018-12-08

**Authors:** Hwan Lim, Jun Sang Ahn, Min Ji Kim, Gi Hwal Son, Joong Kook Park, Jae Yoon Shim, Il Young Kim, Ji Hyung Kim, Sung Myoun Cho, Eung Gi Kwon, Jong Suh Shin, Byung Ki Park

**Affiliations:** 1 Hanwoo Research Institute, National Institute of Animal Science, RDA, Pyeongchang, 25340 South Korea; 2 College of Animal Life Sciences, Kangwon National University, Chuncheon, 24341 South Korea; 3 Nonghyup Feed Co., LTD, Seoul, 05398 South Korea

**Keywords:** Hanwoo, Weaning, Castration, Growth performance, Carcass characteristics

## Abstract

**Background:**

Recently, as production costs have been increasing owing to rising feed prices worldwide, shortening the age of slaughter has been recognized as a way to increase farm income. In Korea, the raising period for Hanwoo steers is over 31 months with the delay of weaning and castration stated as one of the reasons for the increase in the raising period. Thus far, studies on age of weaning and castration have been conducted individually, and there have been no studies on the combined effects of weaning and castration ages on the growth performance and carcass characteristics in Hanwoo steers.

**Methods:**

Weaning ages were calculated at 80 or 130 days of age, and castration ages were calculated at 90 days and 180 days of age. Calves were allocated to one of the four treatment groups: W80C90 (weaning at 80 days of age and castration at 90 days of age), W80C180, W130C90, and W130C180.

**Results:**

For the entire experimental period, weaning and castration ages did not significantly affect growth performance of Hanwoo steers. In addition, weaning and castration ages did not affect the overall yield and quality traits of carcass in Hanwoo steers.

**Conclusion:**

Weaning and castration ages had small effects on growth performance and carcass characteristics in Hanwoo steers. Therefore, the early weaning and castration ages are recommended to reduce the slaughter age without any negative effects on meat quality grade.

## Background

The slaughter age can be changed depending on the age of weaning and castration, sex, nutrition, genetic traits, feed costs, and price of carcasses [20, 27]. Recently, as production costs have been increasing owing to rising feed prices worldwide, shortening the age of slaughter has been recognized as a way to increase farm income. Therefore, it is necessary to look for ways to shorten the age of slaughter while maintaining growth performance and meat quality.

In the Korea feeding standard [23], 90 days of age is suggested as a suitable weaning age; however, there are still many farms that do not wean until after 120 days of age. In addition, the study of the optimal weaning age for Hanwoo calves has been conducted mainly on the body weight gain only until the growing period [23].

The optimal age of castration for Hanwoo calves is recommended before 8 months of age; however, many farms are conducting castration from 4 to 14 months of age. In Korea, the raising period for Hanwoo steers is over 31 months [4, 16, 28] with the delay of weaning and castration stated as one of the reasons for the increase in the raising period. Thus far, studies on age of weaning and castration have been conducted individually, and there have been no studies on the combined effects of weaning and castration ages on the growth performance and carcass grade in Hanwoo steers.

It is reported that shortening of the weaning age is advantageous for reproductive efficiency of calving cows [17, 22], growth performance of calves [3, 11, 25], improving carcass quality [19, 21], and profitability of steers [32].

Even if castration is recommended for producing high-quality meat [35], the optimal age of castration has not been clearly defined. In general, the delay of castration is favorable for average daily gain (ADG) [10, 34], feed efficiency [2, 6], and carcass weight [26, 30]; however, it has been reported to be disadvantageous for meat quality characteristics such as marbling [5, 9, 13] and texture [26, 30]. To prevent deterioration of carcass quality (meat quality grade) due to castration delay, an additional fattening period is required. Thus, early castration is advantageous if the aim is to shorten the slaughter age while maintaining the marbling score and meat quality grade.

The present study was undertaken to investigate the effects of weaning and castration ages on the growth performance, blood metabolites, and carcass characteristics of Hanwoo steers. Additionally, the study was conducted to identify the optimal weaning and castration ages that can maintain meat quality while shortening the slaughter age by responding to the price decrease of high class meat or the increase of international grain prices.

## Methods

### Experimental period and animals

The present study was conducted using different weaning and castration ages over almost 3 years, from March 2015 to November 2017. Forty-eight Hanwoo calves were delivered sequentially from March to April 2015 for approximately 35 days at a farm in Anseong, Gyeonggi-do.

### Treatments and management

Weaning ages (W) were calculated at 80 or 130 days of age, and castration ages (C) were calculated at 90 days and 180 days of age. In the present study, the early weaning and castration ages were determined to be 80 and 90 days of age, respectively, because weaning and castration at many Hanwoo farms are conventionally practiced after 120 (4 months of age) and 180 days (6 months of age), respectively. Calves were allocated to one of the four treatment groups: W80C90 (weaning at 80 days of age and castration at 90 days of age), W80C180 (weaning at 80 days of age and castration at 180 days of age), W130C90 (weaning at 130 days of age and castration at 90 days of age), and W130C180 (weaning at 130 days of age and castration at 180 days of age). Weaning and castration ages were determined by calculating the ordered average birth date of 12 out of 48 bull calves born on March 1, 2015. The mean births of W80C90, W80C180, W130C90, and W130C180 were March 9 (± 2.9 days), March 20 (± 2.8 days), March 26 (± 1.4 days), and April 5 (± 3.8 days), respectively. The castrations were performed under surgical procedure. Calves of W130C90 were treated and stabilized in an independent space for approximately one day after castration, and then placed in pens with their mother.

Six calves were allocated per pen (5 × 10 m) with sawdust to a thickness of approximately 20 cm. Concentrate was fed three times daily (08:30, 13:00, and 17:00) using an automatic feeding system. Concentrate was restrictively fed at 1.7% of body weight (as-fed basis) from the growing to early fattening periods and was restrictively fed at 1.3% of body weight for the late fattening period. Rice straw (dry matter 90.18%, crude protein 3.65%, ether extract 1.02%, crude fiber 34.19%, neutral detergent fiber 70.21%, acid detergent fiber 38.13%, crude ash 10.58%, Ca 0.09%, and P 0.05%) was fed in fixed amounts based on months of age. Water could be accessed freely. The formula percentages and nutrient contents of the experimental diets are presented in Table 1.

**Table 1:** Formula and chemical composition of experimental diets (as-fed basis)

Item	Calf starter	Growing	Early fattening	Late fattening
	Formula (%)			
Corn grain	5.00	27.0	35.6	37.0
Wheat grain	9.90	8.0	9.0	17.6
Milk replacer	10.15	–	–	–
Cane molasses	–	4.5	4.0	5.3
Tapioca residue	–	2.9	4.0	5.3
Wheat flour	–	1.0	1.0	1.0
Rapeseed meal	–	6.0	0.8	–
Whey powder	10.00	–	–	–
Soybean hull	10.00	–	–	–
Wheat bran	14.00	7.0	5.0	5.0
Corn gluten feed	10.00	13.0	14.0	15.0
Isolated soy protein	5.50	–	–	–
Distillers grain	–	8.1	–	–
Coconut meal	–	6.0	5.0	1.8
Palm kernel meal	–	12.0	10.0	6.0
Banana meal	10.00	–	–	–
Beet pulp	8.00	–	–	–
Soybean oil	3.00	0.1	0.1	0.1
Limestone (1 mm)	1.85	2.4	1.7	1.5
Salt dehydrate	0.50	0.5	0.5	0.5
DCP	0.40	–	–	–
Calf mix (additive)	1.70	–	–	–
	Chemical composition (%)			
Dry matter	89.80	88.67	88.50	87.71
Crude protein	24.30	14.10	12.16	12.00
Ether extract	7.56	3.69	4.33	3.47
Crude fiber	4.72	7.30	6.82	6.71
NDF^1^	27.13	26.51	24.79	23.76
Ca	1.30	1.13	0.80	0.85
P	0.69	0.45	0.39	0.35
Crude ash	8.39	7.89	5.79	5.30
TDN	84.50	70.41	72.92	73.32

*DCP* dicalcium phosphate*, NDF* neutral detergent fiber*, TDN* total digestible nutrients

### Measurements and analysis

Average daily gain (ADG) was calculated by measuring body weight at 10 am every 2 months. Feed intake was measured weekly and the amount of feed intake was determined by measuring the orts present before morning feeding. The feed conversion ratio (FCR) was calculated using dry matter intake (DMI) and ADG.

Blood samples were taken at 2 monthly intervals from the jugular vein of the experimental animals. A 3 ml sample of blood for analysis of blood chemical values was collected using an 18-gauge needle and a vacuum blood vessel (Vacutainer; Becton- Dickinson, NJ) coated with anticoagulant. The collected blood was stored in an ice box and transferred to the laboratory within 6 h. The blood was centrifuged at 3000×*g* for 10 min to separate the serum and was analyzed using an automatic blood analyzer (Hitachi 7020, Hitachi Ltd., Tokyo, Japan). Factors measured included glucose, total cholesterol, albumin, total protein, triglyceride, total bilirubin, blood urea nitrogen (BUN), gamma-glutamyl transpeptidase (GGT), glutamic oxaloacetic transaminase (GOT), glutamic-pyruvic transaminase (GPT), non-esterified fatty acid (NEFA), creatinine, calcium (Ca), phosphorus (P), and magnesium (Mg). Chemical compositions of the experimental diets were analyzed based on the method described by AOAC [1] and Van Soest [33].

At the end of the experimental period, all steers were slaughtered at the local slaughterhouse to assess carcass yield and quality grades. Carcass traits were determined at the 13th rib section from the left side of each carcass and graded by meat graders using the criteria described by the Korean carcass grading system [14]. Meat quality traits were measured for marbling score, meat color, fat color, texture, and maturity, and yield traits were measured for carcass weight, back fat thickness, and rib eye area. Carcass yield index (YI) was calculated based on the following equation: YI = [68.184 - (0.625 × back fat thickness, mm) + (0.130 × rib eye area, cm^2^) - (0.024 × carcass weight, kg)].

From this equation, scores over 67.50 were rated as A (best), scores of 62.00–67.50 were rated as B, and scores less than 62.00 were rated as C (worst). The meat quality grades were 1^++^ grade (best) for 8 and 9, 1^+^ grade for 6 and 7, 1 grade for 4 and 5, 2 grade for 2 and 3, and 3 grade (worst) for 1. For the statistical analysis of the yield grade, 3 points were given for yield grade A, 2 points for B grade, and 1 point for C grade. In the case of the quality grade, 5 points were given for quality grade 1^++^, 4 points for 1^+^ grade, 3 points for 1 grade, 2 points for 2 grade, and 1 point for 3 grade.

### Statistical analysis

In the present study, to estimate the environmental effects on body weight, ADG, and blood metabolite concentration based on weaning and castration ages, the following linear model was used for least squares analysis: y_ijkl_ = μ + TRT_i_ + β_1_X_1ij_ + β_2_X_2ik_ + e_ijkl_, where, μ = overall average, TRT_i_ = effect of treatment (1–4), X_1_, X_2_ = covariation of castration age and measurement month, β_1_, β_2_ = regression coefficient, and e_ijkl_ = random error effect.

The least squares method was performed to estimate environmental effects on body weight, ADG, feed intake, FCR, and concentration of blood metabolites by combining the treatment groups (same weaning and castration ages): y_ij_ = μ + TRT_i_ + e_ij_, where, μ = overall average, TRT_i_ = effect of treatment (1–4), and e_ij_ = random error effect.

The linear model was analyzed using SAS 9.1 [29] software package and the variance analysis was performed using Type III squared fit for unbalanced data among the four squares presented in the SAS/generalized linear model analysis. The statistical significance differences for the treatments between the least squares averages were tested with the following null hypothesis at significance level of 5%: Ho: LSM(i) = LSM(j), where, LSM (i(j)) is the least squares average of the I (j) the effects (I ≠ j).

All results of the present study were analyzed by *t*-tests using the least significant difference procedure of the SAS package program (release. 9.1.3 version, 2005). The statistically significant differences of feed intake, body weight, and concentration of blood metabolites between groups were analyzed following the generalized linear model: Y_ij_ = μ + TRT_i_ + e_ij_, where, μ = overall average, TRT_i_ = effect of treatment (1–4), and e_ij_ = random error effect.

## Results and discussion

### Growth performance

For the growing period, ADG was lower in W130C180 than that of the other treatment groups (Table 2); however, there was no statistically significant difference. FCR was lower in W80C180 than that of W130C180 (*P* < 0.05); however, results were similar between the other treatment groups. For the early and late fattening periods, ADGs were higher in W80C90 than that of the other treatment groups; however, there was no statistically significant difference. FCRs were lower in W80C90 than that of the other treatment groups. For the entire experimental period, there were no effects of the weaning and castration ages on ADG and FCR of Hanwoo steers. There were no differences in concentrate, rice straw, and dry matter intakes between treatment groups because concentration and rice straw were restricted by the weight of steers over the entire experimental period.

**Table 2:** Effects of weaning and castration ages on growth performance of Hanwoo steers

Item	Treatment^c^				*P*-value
	W80C90	W80C180	W130C90	W130C180	
	Growing period (3–12 months of age)				
Initial body weight (kg)	112.7 ± 12.3	114.9 ± 13.8	118.7 ± 10.8	110.7 ± 13.7	0.062
Final body weight (kg)	314.6 ± 31.3	317.5 ± 29.2	320.2 ± 24.0	299.0 ± 23.8	0.277
Average daily gain (kg)	0.74 ± 0.08	0.75 ± 0.08	0.74 ± 0.07	0.70 ± 0.06	0.362
Concentrate (DM, kg)	3.58 ± 0.58	3.58 ± 0.58	3.58 ± 0.58	3.58 ± 0.58	–
Rice straw (DM, kg)	1.78 ± 0.25	1.78 ± 0.25	1.78 ± 0.25	1.78 ± 0.25	–
Dry matter intake (kg)	5.36 ± 0.57	5.36 ± 0.57	5.36 ± 0.57	5.36 ± 0.57	–
Feed conversion ratio	7.24^ab^ ± 0.60	6.96^b^ ± 0.65	7.24^ab^ ± 0.51	8.68^a^ ± 0.53	0.011
	Early fattening period (13–22 months of age)				
Initial body weight (kg)	314.6 ± 31.3	317.5 ± 29.2	320.2 ± 24.0	299.0 ± 23.8	0.277
Final body weight (kg)	569.5 ± 40.5	584.7 ± 56.2	583.8 ± 57.8	570.7 ± 48.9	0.828
Average daily gain (kg)	0.84 ± 0.05	0.88 ± 0.11	0.87 ± 0.12	0.90 ± 0.13	0.500
Concentrate (DM, kg)	5.82 ± 1.11	5.82 ± 1.11	5.82 ± 1.11	5.82 ± 1.11	–
Rice straw (DM, kg)	2.39 ± 0.21	2.39 ± 0.21	2.39 ± 0.21	2.39 ± 0.21	–
Dry matter intake (kg)	8.21 ± 0.45	8.21 ± 0.45	8.21 ± 0.45	8.21 ± 0.45	–
Feed conversion ratio	10.06 ± 0.77	9.70 ± 1.41	9.71 ± 1.31	9.41 ± 1.59	0.714
	Late fattening period (23–31 months of age)				
Initial body weight (kg)	569.5 ± 40.5	584.7 ± 56.2	583.8 ± 57.8	570.7 ± 48.9	0.838
Final body weight (kg)	756.5 ± 60.1	783.0 ± 79.3	781.8 ± 75.6	766.1 ± 72.9	0.793
Average daily gain (kg)	0.65 ± 0.09	0.69 ± 0.12	0.69 ± 0.08	0.68 ± 0.17	0.868
Concentrate (DM, kg)	7.97 ± 0.81	8.13 ± 0.64	8.13 ± 0.64	8.13 ± 0.64	–
Rice straw (DM, kg)	0.96 ± 0.34	0.96 ± 0.34	0.96 ± 0.34	0.96 ± 0.34	–
Dry matter intake (kg)	8.93 ± 0.83	9.09 ± 0.64	9.10 ± 0.64	9.10 ± 0.64	–
Feed conversion ratio	13.77 ± 3.93	11.98 ± 2.03	11.87 ± 1.66	12.10 ± 2.39	0.642
	Entire period (3–31 months of age)				
Initial body weight (kg)	112.7 ± 12.3	114.9 ± 13.8	118.7 ± 10.8	110.7 ± 13.7	0.062
Final body weight (kg)	756.5 ± 60.1	783.0 ± 79.3	781.8 ± 75.6	766.1 ± 72.9	0.793
Average daily gain (kg)	0.75 ± 0.06	0.78 ± 0.07	0.77 ± 0.05	0.77 ± 0.08	0.739
Concentrate (DM, kg)	5.72 ± 2.33	5.78 ± 2.37	5.75 ± 2.43	5.75 ± 2.43	–
Rice straw (DM, kg)	1.73 ± 0.65	1.73 ± 0.65	1.69 ± 0.67	1.69 ± 0.67	–
Dry matter intake (kg)	7.45 ± 2.17	7.50 ± 2.19	7.45 ± 2.33	7.45 ± 2.33	–
Feed conversion ratio	10.2 ± 4.09	9.35 ± 3.05	9.26 ± 3.11	9.30 ± 3.31	0.879

Means ± standard deviation

^a,b^Means with different superscripts within the same row are significantly different (*P* < 0.05)

^c^W80C90: weaning at 80 days of age and castration at 90 days of age; W80C180: weaning at 80 days of age and castration at 180 days of age; W130C90: weaning at 130 days of age and castration at 90 days of age; W130C180: weaning at 130 days of age and castration at 180 days of age

For the growing period, ADG was lower in W130 than that of W80 (Table 3); however, there was no statistically significant difference. There were no effects of weaning age on ADG, feed intake, and FCR over the entire experimental period.

**Table 3:** Effects of weaning age on growth performance of Hanwoo steers

Item	Weaning age^a^		SE	Pr > |t|
	W80	W130		
	Growing period (3–12 months of age)			
Initial body weight (kg)	110.3 ± 13.3	111.5 ± 14.3	3.816	0.758
Final body weight (kg)	316.1 ± 30.3	309.6 ± 26.1	8.807	0.472
Average daily gain (kg)	0.75 ± 0.08	0.72 ± 0.06	0.023	0.237
Concentrate (DM, kg)	3.58 ± 0.58	3.58 ± 0.58	–	–
Rice straw (DM, kg)	1.78 ± 0.25	1.78 ± 0.25	–	–
Dry matter intake (kg)	5.36 ± 0.57	5.36 ± 0.57	–	–
Feed conversion ratio	7.15 ± 0.63	7.44 ± 0.53	0.180	0.269
	Early fattening period (13–22 months of age)			
Initial body weight (kg)	316.1 ± 30.3	309.6 ± 26.1	8.807	0.472
Final body weight (kg)	577.1 ± 49.6	577.3 ± 53.9	14.282	0.857
Average daily gain (kg)	0.86 ± 0.09	0.89 ± 0.13	0.032	0.248
Concentrate (DM, kg)	5.82 ± 1.11	5.82 ± 1.11	–	–
Rice straw (DM, kg)	2.39 ± 0.21	2.39 ± 0.21	–	–
Dry matter intake (kg)	8.21 ± 0.45	8.21 ± 0.45	–	–
Feed conversion ratio	9.88 ± 1.15	9.56 ± 1.47	0.355	0.378
	Late fattening period (23–31 months of age)			
Initial body weight (kg)	577.10 ± 50.63	577.27 ± 55.07	52.900	0.991
Final body weight (kg)	769.77 ± 73.13	773.94 ± 76.28	74.721	0.848
Average daily gain (kg)	0.67 ± 0.12	0.69 ± 0.14	0.128	0.707
Concentrate (DM, kg)	8.05 ± 0.71	8.13 ± 0.62	–	–
Rice straw (DM, kg)	0.96 ± 0.33	0.96 ± 0.33	–	–
Dry matter intake (kg)	9.01 ± 0.73	9.10 ± 0.62	–	–
Feed conversion ratio	12.87 ± 3.09	11.99 ± 1.95	2.586	0.454
	Entire period (3–31 months of age)			
Initial body weight (kg)	110.3 ± 13.3	111.5 ± 14.3	14.100	0.772
Final body weight (kg)	769.8 ± 71.6	773.9 ± 74.7	74.721	0.848
Average daily gain (kg)	0.77 ± 0.07	0.77 ± 0.08	0.079	0.881
Concentrate (DM, kg)	5.75 ± 2.33	5.75 ± 2.41	–	–
Rice straw (DM, kg)	1.73 ± 0.65	1.69 ± 0.67	–	–
Dry matter intake (kg)	7.47 ± 2.17	7.45 ± 2.31	–	–
Feed conversion ratio	9.77 ± 3.56	9.28 ± 3.14	3.358	0.601

Means ± standard deviation

^a^W80: weaning at 80 days of age at weaning; W130: weaning at 130 days of age

For the growing period, there were no differences in ADG and FCR based on the castration age (Table 4). Final body weight was 5.4 kg higher in C180 than that in C90; however, there was no statistically significant difference. There were no effects of castration age on ADG, feed intake, and FCR over the entire experimental period.

**Table 4:** Effects of castration age on growth performance of Hanwoo steers

Item	Castration age^a^		SE	Pr > |t|
	C90	C180		
	Growing period (3–12 months of age)			
Initial body weight (kg)	115.7 ± 12.2	112.8 ± 14.2	3.511	0.072
Final body weight (kg)	317.4 ± 28.8	308.3 ± 28.8	8.447	0.292
Average daily gain (kg)	0.74 ± 0.07	0.73 ± 0.08	0.023	0.914
Concentrate (DM, kg)	3.58 ± 0.58	3.58 ± 0.58	–	–
Rice straw (DM, kg)	1.78 ± 0.25	1.78 ± 0.25	–	–
Dry matter intake (kg)	5.36 ± 0.57	5.36 ± 0.57	–	–
Feed conversion ratio	7.24 ± 0.57	7.24 ± 0.64	0.188	0.991
	Early fattening period (13–22 months of age)			
Initial body weight (kg)	317.4 ± 28.8	308.3 ± 28.8	8.447	0.292
Final body weight (kg)	576.6 ± 50.4	577.7 ± 53.1	13.293	0.991
Average daily gain (kg)	0.86 ± 0.10	0.89 ± 0.12	0.031	0.224
Concentrate (DM, kg)	5.82 ± 1.11	5.82 ± 1.11	–	–
Rice straw (DM, kg)	2.39 ± 0.21	2.39 ± 0.21	–	–
Dry matter intake (kg)	8.21 ± 0.45	8.21 ± 0.45	–	–
Feed conversion ratio	9.88 ± 1.11	9.56 ± 1.54	0.350	0.351
	Late fattening period (23–31 months of age)			
Initial body weight (kg)	576.65 ± 51.52	577.72 ± 54.24	52.897	0.944
Final body weight (kg)	769.15 ± 70.94	774.56 ± 78.28	74.700	0.803
Average daily gain (kg)	0.67 ± 0.09	0.69 ± 0.16	0.128	0.682
Concentrate (DM, kg)	8.05 ± 0.71	8.13 ± 0.62	–	–
Rice straw (DM, kg)	0.96 ± 0.33	0.96 ± 0.33	–	–
Dry matter intake (kg)	9.01 ± 0.73	9.1 ± 0.62	–	–
Feed conversion ratio	12.82 ± 3.02	12.04 ± 2.09	2.595	0.511
	Entire period (3–31 months of age)			
Initial body weight (kg)	115.7 ± 12.2	112.8 ± 14.2	13.241	0.076
Final body weight (kg)	769.2 ± 70.9	774.6 ± 78.3	74.700	0.803
Average daily gain (kg)	0.76 ± 0.07	0.78 ± 0.08	0.079	0.449
Concentrate (DM, kg)	5.74 ± 2.36	5.76 ± 2.38	–	–
Rice straw (DM, kg)	1.71 ± 0.66	1.71 ± 0.66	–	–
Dry matter intake (kg)	7.45 ± 2.23	7.47 ± 2.24	–	–
Feed conversion ratio	9.73 ± 3.59	9.33 ± 3.11	3.361	0.665

Means ± standard deviation

^a^C90: castration at 90 days of age; C180: castration at 180 days of age

In the present study, there were no overall effects of weaning and castration ages on ADG, feed intake, and FCR of Hanwoo steers; however, the final body weight was considered to be slightly advantageous in the customary weaning and castration ages than that in the early weaning and castration ages. Previous studies [24, 25] reported that the early weaned calves had higher ADG. However, ADG tended to be slightly higher for the growing period owing to the early weaning in the present study, yet they were similar between the other treatment groups over the entire experimental period. These results are caused by differences in calf management methods, experimental periods (weaning to growing vs. weaning to late fattening), and feeding method of concentrate (ad libitum or restricted feeding).

Kwon et al. [15] reported that there was no effect of the weaning ages (90 and 120 days) on concentrates, rice straw, dry matter, crude protein, and TDN intakes, which is in agreeance with our results. In the present study, there was no difference in feed intake until 3 months of age, as the amount of milk suckling was limited to two times per day from 1 month after birth. The results of no significant difference in feed intake were due to restricted feeding of concentrates (1.7% of BW for growing and early fattening periods and 1.3% of BW for late fattening period) and fixed feeding of rice straw by months of age. In the present study, the purpose of limited milk suckling was to increase concentrate and hay intake considering the rapid increase in energy demand of calves from 3 weeks of age and the rapid decrease in milk yield of mother cows [11]. Therefore, the ADG of calves was not lower than that of the early weaning age if the amount of milk suckling is restricted from 1 month after birth. This result would be useful to share with farmers for incorporation into their management systems.

In the present study, we did not investigate return to estrus, services per conception, and calving interval based on the weaning age. However, it is desirable to shorten the weaning age as much as possible, considering shortening of return to estrous [31] and improvement of reproductive efficiency [8, 17, 24].

In the experimental design process of the present study, the early castration was predicted to be disadvantageous to ADG and FCR compared to the customary castration age. However, from the results, there was no effect of castration age on ADG and FCR, which appears to be related to castration age and feeding methods. Regardless of the treatment group, there was no significant difference in stress between the treatment groups as all calves were castrated before puberty. In addition, although there was a slight difference in ADG and FCR over the growing period, there was no difference in ADG and FCR based on the castration age over the entire experimental period because there was similar feed intake owing to the restricted feeding of concentrate.

In general, delayed castration is known as being advantageous to ADG [10, 34] and feed efficiency [2, 6]. However, Worrell et al. [35] reported that early castration before puberty did not reduce ADG. This finding supports the results of the present study. In addition, previous studies [5, 7] reported similar results as the present study that there was no difference in ADG in relation to the castration age. When castration is delayed, increased stress [12, 13] occurs and the extension of the fattening period [5, 13] is inevitable to produce the same meat quality grade. Therefore, early castration is advantageous to shorten the age of slaughter taking into consideration the meat quality grade.

### Blood metabolites

There were no effects of weaning and castration ages on concentrations of albumin, BUN, total protein, glucose, creatinine, cholesterol, total glyceride, NEFA, Ca, P, GOT, GPT, and GGT of Hanwoo steers (Table 5).

**Table 5:** Effects of weaning and castration ages on metabolites of Hanwoo steers

Item	Treatment^a^				SE	*P*-value
	W80C90	W80C180	W130C90	W130C180		
	Growing period (3–12 months of age)					
Albumin (g/dl)	3.43	3.55	3.63	3.53	0.142	0.225
BUN (mg/dl)	13.23	12.98	13.26	12.46	1.248	0.353
Total protein (g/dl)	6.24	6.54	6.69	6.45	0.337	0.321
Glucose (mg/dl)	62.83	62.83	65.58	68.75	11.255	0.311
Creatinine (mg/dl)	0.98	1.00	1.07	1.05	0.124	0.235
Cholesterol (mg/dl)	126.08	113.83	128.67	115.58	24.226	0.105
Total glyceride (mg/dl)	24.52	24.33	24.42	22.65	2.561	0.325
NEFA (μEq/L)	107.92	149.42	141.92	135.83	27.331	0.289
Calcium (mg/dl)	9.59	9.66	9.23	9.14	0.318	0.335
Phosphorus (mg/dl)	8.60	8.77	8.73	8.57	0.614	0.312
GOT (IU/L)	68.50	62.92	60.42	62.33	7.203	0.114
GPT (IU/L)	25.77	24.56	22.82	24.15	5.365	0.235
GGT (mg/dl)	22.67	19.08	19.75	19.92	3.217	0.164
	Early fattening period (13–22 months of age)					
Albumin (g/dl)	3.98	4.38	4.21	3.98	0.149	0.055
BUN (mg/dl)	12.70	11.45	13.00	10.93	0.388	0.160
Total protein (g/dl)	8.21	8.50	8.42	8.27	0.222	0.748
Glucose (mg/dl)	60.13	63.29	59.92	66.22	2.120	0.386
Creatinine (mg/dl)	1.16	1.36	1.33	1.27	0.073	0.268
Cholesterol (mg/dl)	171.58	132.42	147.85	157.63	12.299	0.109
Total glyceride (mg/dl)	17.08	18.75	16.50	19.24	1.358	0.506
NEFA (μEq/L)	244.42	291.25	331.46	319.39	19.539	0.162
Calcium (mg/dl)	9.30	9.50	9.30	8.46	0.182	0.179
Phosphorus (mg/dl)	7.87	7.52	7.90	8.28	0.950	0.404
GOT (IU/L)	63.13	56.29	70.00	52.67	5.813	0.337
GPT (IU/L)	27.55	26.92	28.10	34.42	3.526	0.876
GGT (mg/dl)	22.25	19.79	27.39	24.84	2.407	0.135
	Late fattening period (23–31 months of age)					
Albumin (g/dl)	4.26	4.45	4.40	4.36	0.242	0.343
BUN (mg/dl)	16.74	17.40	17.65	16.76	3.053	0.966
Total protein (g/dl)	8.94	9.00	8.98	9.01	0.501	0.783
Glucose (mg/dl)	92.50	92.88	95.83	104.38	6.462	0.106
Creatinine (mg/dl)	1.56	1.54	1.53	1.54	0.176	0.843
Cholesterol (mg/dl)	179.88	164.38	178.17	189.88	32.915	0.869
Total glyceride (mg/dl)	39.63	34.00	47.50	38.00	10.654	0.086
NEFA (μEq/L)	132.50	102.38	127.83	114.25	62.398	0.323
Calcium (mg/dl)	10.08	8.91	9.85	9.95	1.633	0.365
Phosphorus (mg/dl)	8.08	7.80	8.17	7.95	0.668	0.321
GOT (IU/L)	97.00	97.25	97.33	84.00	6.356	0.497
GPT (IU/L)	20.25	18.13	19.17	19.75	3.827	0.542
GGT (mg/dl)	80.88	57.50	59.00	40.13	4.372	0.069

*BUN* blood urea nitrogen*, NEFA* non-esterified fatty acid*, GOT* glutamic oxaloacetic transaminase*, GPT* glutamic-pyruvic transaminase*, GGT,* gamma-glutamyl transpeptidase

^a^W80C90: weaning at 80 days of age and castration at 90 days of age; W80C180: weaning at 80 days of age and castration at 180 days of age; W130C90: weaning at 130 days of age and castration at 90 days of age; W130C180: weaning at 130 days of age and castration at 180 days of age

There were no effects of weaning and castration ages on concentrations of blood metabolites, which was due to no difference in feed intake between the treatment groups in the present study (Tables 2, 3, and 4). In the present study, there was no difference in concentration of blood metabolites owing to similar nutrient supply and utilization efficiency between the treatment groups because of castration before puberty, limited feeding of concentrates, and fixed feeding of rice straw.

### Carcass characteristics

The carcass weight tended to be lower in W80C90 than that of the other treatment groups (Table 6). The rib eye area was wider in W130C90 than that of the other treatment groups; however, there was no statistically significant difference. There were only small effects of weaning and castration ages on the overall yield traits (carcass weight, back fat thickness, rib eye area, yield index, and yield grade score) of Hanwoo steers. The marbling and meat quality scores were higher in W130C90 than that of the other treatment groups; however, there was no statistically significant difference. There were no effects of weaning and castration ages on meat color, fat color, and texture.

**Table 6:** Effects of weaning and castration ages on carcass characteristics of Hanwoo steers

Item	Treatment^a^				*P*-value
	W80C90	W80C180	W130C90	W130C180	
Yield traits^b^					
Carcass weight (kg)	446.33 ± 39.09	470.11 ± 43.4	464.82 ± 40.24	468.58 ± 51.28	0.547
Back fat thickness (mm)	12.08 ± 3.15	13.44 ± 4.36	14.36 ± 3.96	11.50 ± 3.61	0.270
Rib eye area (cm^2^)	93.42 ± 11.8	94.33 ± 6.06	101.45 ± 10.43	97.00 ± 9.84	0.246
Yield index	65.39 ± 1.87	63.99 ± 3.09	64.47 ± 3.56	65.59 ± 3.32	0.579
Yield grade score^c^	2.00 ± 0.60	1.67 ± 0.71	2.00 ± 0.77	2.08 ± 0.67	0.558
Quality traits^d^					
Marbling score	6.58 ± 1.51	6.78 ± 1.30	7.36 ± 1.03	6.33 ± 1.50	0.327
Meat color	4.92 ± 0.67	4.78 ± 0.67	4.91 ± 0.3	4.75 ± 0.62	0.861
Fat color	2.50 ± 0.52	2.43 ± 0.50	2.86 ± 0.49	2.75 ± 0.45	0.086
Texture	1.17 ± 0.39	1.00 ± 0.00	1.00 ± 0.00	1.08 ± 0.29	0.363
Quality grade score^e^	4.08 ± 0.79	4.22 ± 0.67	4.36 ± 0.50	4.08 ± 0.67	0.716

Means ± standard deviation

^a^W80C90: weaning at 80 days of age and castration at 90 days of age; W80C180: weaning at 80 days of age and castration at 180 days of age; W130C90: weaning at 130 days of age and castration at 90 days of age; W130C180: weaning at 130 days of age and castration at 180 days of age

^b^Area was measured from *longissmus* muscle taken at 13th rib and back fat thickness was also measured at 13th rib; Yield index was calculated using the following equation: 68.184 - (0.625 × back fat thickness (mm)) + (0.130 × rib eye area (cm^2^)) - (0.024 × dressed weight amount (kg)); Carcass yield grades from C (low yield) to A (high yield)

^c^A grade = 3, B grade = 2, C grade = 1

^d^Grading ranges are 1 to 9 for marbling score with higher numbers for better quality (1 = devoid, 9 = abundant); meat color (1 = bright red, 7 = dark red); fat color (1 = creamy white, 7 = yellowish); texture (1 = soft, 3 = firm); quality grades from 3 (low quality) to 1^++^ (very high quality)

^e^1^++^ grade = 5, 1^+^ grade = 4, 1 grade = 3, 2 grade = 2, 3 grade = 1

Although there was no statistically significant difference, carcass weight (456.52 vs. 466.78 kg), rib eye area (93.81 vs. 99.13 cm^2^), and meat quality grade score (1.86 vs. 2.04) was higher in W130 than that in W80, respectively (Table 7). The marbling score, meat color, fat color, texture, and meat quality score were similar between the treatment groups.

**Table 7:** Effects of weaning age on carcass characteristics of Hanwoo steers

Item	Weaning age^a^		SE	*p*-value
	W80	W130		
Yield traits^b^				
Carcass weight (kg)	456.52 ± 41.7	466.78 ± 45.33	43.639	0.440
Back fat thickness (mm)	12.67 ± 3.68	12.87 ± 3.97	3.834	0.862
Rib eye area (cm^2^)	93.81 ± 9.57	99.13 ± 10.15	9.880	0.082
Yield index	64.79 ± 2.50	65.05 ± 3.40	3.007	0.774
Yield grade score^c^	1.86 ± 0.65	2.04 ± 0.71	0.682	0.370
Quality traits^d^				
Marbling score	6.67 ± 1.39	6.83 ± 1.37	1.380	0.704
Meat color	4.86 ± 0.65	4.83 ± 0.49	0.575	0.859
Fat color	2.53 ± 0.51	2.77 ± 0.34	0.430	0.082
Texture	1.10 ± 0.30	1.04 ± 0.21	0.257	0.508
Quality grade score^e^	4.14 ± 0.73	4.22 ± 0.60	0.663	0.712

Means ± standard deviation

^a^W80C90: weaning at 80 days of age and castration at 90 days of age; W80C180: weaning at 80 days of age and castration at 180 days of age; W130C90: weaning at 130 days of age and castration at 90 days of age; W130C180: weaning at 130 days of age and castration at 180 days of age

^b^Area was measured from *longissmus* muscle taken at 13th rib and back fat thickness was also measured at 13th rib; Yield index was calculated using the following equation: 68.184 - (0.625 × back fat thickness (mm)) + (0.130 × rib eye area (cm^2^)) - (0.024 × dressed weight amount (kg)); Carcass yield grades from C (low yield) to A (high yield)

^c^A grade = 3, B grade = 2, C grade = 1

^d^Grading ranges are 1 to 9 for marbling score with higher numbers for better quality (1 = devoid, 9 = abundant); meat color (1 = bright red, 7 = dark red); fat color (1 = creamy white, 7 = yellowish); texture (1 = soft, 3 = firm); quality grades from 3 (low quality) to 1^++^ (very high quality)

^e^1^++^ grade = 5, 1^+^ grade = 4, 1 grade = 3, 2 grade = 2, 3 grade = 1

The carcass weight tended to be higher in C180 (469.24 kg) than that of C90 (455.17 kg); however, there was no statistically significant difference (Table 8). Although there was no statistically significant difference, the back fat thickness (13.17 vs. 12.33 mm) and rib eye area (97.26 vs. 95.86 cm^2^) was higher in C90 than that of C180, respectively. The marbling score (6.96 vs. 6.52) and meat quality score (4.22 vs. 4.14) was higher in C90 than that in C180, respectively; however, there was no statistically significant difference.

**Table 8:** Effects of castration age on carcass characteristics of Hanwoo steers

Item	Castration age^a^		SE	*p*-value
	C90	C180		
Yield traits^b^				
Carcass weight (kg)	455.17 ± 39.86	469.24 ± 46.91	43.361	0.289
Back fat thickness (mm)	13.17 ± 3.66	12.33 ± 3.97	3.811	0.469
Rib eye area (cm^2^)	97.26 ± 11.66	95.86 ± 8.36	10.223	0.652
Yield index	64.95 ± 2.78	64.90 ± 3.24	3.010	0.960
Yield grade score^c^	2.00 ± 0.67	1.90 ± 0.70	0.687	0.648
Quality traits^d^				
Marbling score	6.96 ± 1.33	6.52 ± 1.40	1.364	0.299
Meat color	4.91 ± 0.51	4.76 ± 0.62	0.570	0.385
Fat color	2.74 ± 0.45	2.57 ± 0.51	0.478	0.251
Texture	1.09 ± 0.29	1.05 ± 0.22	0.257	0.615
Quality grade score^e^	4.22 ± 0.67	4.14 ± 0.65	0.663	0.712

Means ± standard deviation

^a^W80C90: weaning at 80 days of age and castration at 90 days of age; W80C180: weaning at 80 days of age and castration at 180 days of age; W130C90: weaning at 130 days of age and castration at 90 days of age; W130C180: weaning at 130 days of age and castration at 180 days of age

^b^Area was measured from *longissmus* muscle taken at 13th rib and back fat thickness was also measured at 13th rib; Yield index was calculated using the following equation: 68.184 - (0.625 × back fat thickness (mm)) + (0.130 × rib eye area (cm^2^)) - (0.024 × dressed weight amount (kg)); Carcass yield grades from C (low yield) to A (high yield)

^c^A grade = 3, B grade = 2, C grade = 1

^d^Grading ranges are 1 to 9 for marbling score with higher numbers for better quality (1 = devoid, 9 = abundant); meat color (1 = bright red, 7 = dark red); fat color (1 = creamy white, 7 = yellowish); texture (1 = soft, 3 = firm); quality grades from 3 (low quality) to 1^++^ (very high quality)

^e^1^++^ grade = 5, 1^+^ grade = 4, 1 grade = 3, 2 grade = 2, 3 grade = 1

In the present study, there were small effects of weaning and castration ages on meat quantity and yield traits of Hanwoo steers. This result was probably due to the influence of calf management, feeding methods, and castration age. First, there was no difference in feed intake until 3 months of age as the amount of milk suckling was limited to two times per day from 1 month after birth. Second, there was no difference in the supply of nutrients (energy, protein, and so on) because feed intake (Tables 2, 3, and 4) was similar between the treatment groups due to restricted feeding of concentrates and fixed feeding of rice straw regardless of the treatment groups. Finally, the level of stress caused by castration was similar because calves were castrated before puberty, regardless of the treatment groups. As a result, it is considered that carcass grades were similar as there was no difference in the nutrient utilization efficiency.

In general, early castration has been known to be disadvantageous to carcass weight [18], yet advantageous to marbling score [5, 9, 13]. Although there was no statistically significant difference, results of the present study showed a decrease in carcass weight and increase in marbling with early castration. These results are similar to previous studies. However, the weaning age was considered together with the castration age in the present study, which is different to previous studies. Although there was no positive effect on carcass quality as in previous studies [19, 22], there was no negative effect of the early weaning on the carcass grade of Hanwoo steers and the reproduction efficiency of mother cows. Therefore, considering these points, early weaning is expected to have a positive influence on calves and mother cows.

Although the data are not presented in the present study, ultrasound results at 26 months of age showed smaller rib eye areas owing to the early weaning but higher rib eye areas and marbling due to the early castration. At 28 months of age, the marbling score by ultrasound increased owing to the early weaning and castration. Considering these ultrasound results, when the slaughter age is adjusted from 31 months to 28–29 months of age, the early weaning and castration is favorable to the rib eye area and marbling score except for carcass weight.

Therefore, the results of the present study suggest that the weaning and castration ages have a small effect on ADG, feed intake, FCR, blood metabolism, and carcass characteristics of Hanwoo steers. However, it is considered that early castration is advantageous when the slaughter age is shortened with marbling reduction (i.e., meat quality grade) minimized. Furthermore, early weaning is favorable when reproductive efficiency of mother cows is considered.

The present findings indicated that weaning and castration ages resulted in similar growth performance and carcass characteristics without any negative effect on marbling score in Hanwoo steers. Therefore, early weaning and castration is recommended to reduce the age of slaughter considering meat quality of Hanwoo steers and reproductive efficiencies of Hanwoo cows.

## Conclusion

For the entire experimental period, weaning and castration ages did not significantly affect average daily gain, dry matter intake, and feed conversion ratio of Hanwoo steers. Rib eye area, marbling, and quality grade scores were slightly but not significantly higher in W130C90 than in the other treatment groups. Weaning and castration ages did not affect the overall yield and quality traits of carcass. The present findings indicated that weaning and castration ages resulted in similar growth performance and carcass characteristics without any negative effect on marbling score in Hanwoo steers. Therefore, early weaning and castration is recommended to reduce the age of slaughter considering meat quality of Hanwoo steers and reproductive efficiencies of Hanwoo cows.

## Abbreviations

ADG: Average daily gain
BUN: Blood urea nitrogen
C: Castration
Ca: Calcium
DCP: Dicalcium phosphate
DMI: Dry matter intake
FCR: Feed conversion ratio
GGT: Gamma-glutamyl transpeptidase
GOT: Glutamic oxaloacetic transaminase
GPT: Glutamic-pyruvic transaminase
Mg: Magnesium
NDF: Neutral detergent fiber
NEFA: Non-esterified fatty acid
P: Phosphorus
TDN: Total digestible nutrients
W: Weaning, YI: Yield index
